# Lipid Nanoparticle-Mediated Lymphatic Delivery of Immunostimulatory Nucleic Acids

**DOI:** 10.3390/pharmaceutics13040490

**Published:** 2021-04-03

**Authors:** Dongyoon Kim, Yina Wu, Gayong Shim, Yu-Kyoung Oh

**Affiliations:** 1 College of Pharmacy and Research Institute of Pharmaceutical Sciences, Seoul National University, Seoul 08826, Korea; mmamic@snu.ac.kr (D.K.); yena-oh@snu.ac.kr (Y.W.); 2 School of Systems Biomedical Science, Soongsil University, Seoul 06978, Korea

**Keywords:** nucleic acid adjuvant, lipid nanoparticle, lymphatic delivery, dendritic cell-targeted delivery

## Abstract

Lymphatic delivery of a vaccine can be achieved using a dendritic cell (DC)-targeted delivery system that can cause DC to migrate to lymph nodes upon activation by an adjuvant. Here, we designed a mannose-modified cationic lipid nanoparticle (M-NP) to deliver the nucleic acid adjuvant, polyinosinic:polycytidylic acid (PIC). PIC-loaded M-NP (PIC/M-NP) showed stable lipoplexes regardless of the ligand ratio and negligible cytotoxicity in bone marrow-derived DC. DC uptake of PIC/M-NP was demonstrated, and an increased mannose ligand ratio improved DC uptake efficiency. PIC/M-NP significantly promoted the maturation of bone marrow-derived DC, and local injection of PIC/M-NP to mice facilitated lymphatic delivery and activation (upon NP uptake) of DC. Our results support the potential of PIC/M-NP in delivering a nucleic acid adjuvant for the vaccination of antigens.

## 1. Introduction

A vaccine adjuvant, which is an immunostimulatory component of a vaccine formulation, is often very important for the successful vaccination of an antigen [1,2,3]. Most recently developed vaccines do not apply the entire pathogen but rather use certain components (e.g., major proteins) as antigens [4]. However, a subunit vaccine alone often fails to induce a strong immune response [5]. Aluminum-containing adjuvants have the longest history of use [6,7], but they have a limited ability to induce cellular immunity and thus are not suitable for intracellular antigen applications [8]. In addition, aluminum adjuvants can have some side effects, raising safety issues [6,9]. Since preventive vaccines are used in people of various ages, safety is a top priority in vaccine development.

Adjuvants composed of nucleic acids have great advantages in terms of biocompatibility and safety [8]. The Toll-like receptor 9-agonizing nucleic acid, CpG 1018, is included as an adjuvant in the hepatitis B vaccine, HEPLISAV-B^®^, which was recently approved by the U.S. FDA [10]. Although this supported the efficacy of nucleic acid adjuvants, the pharmaceutical application of nucleic acids has faced some limitations. For example, many nucleic acid adjuvants target intracellular molecules in immune-stimulating pathways and thus must be introduced into dendritic cells (DC) [8]. In addition, nucleic acids have weak stability due to the action of in vivo nucleases [11]. To compensate for these limitations, nucleic acid adjuvants may need to be applied using an appropriately designed delivery system.

Lipid nanoparticles (LNP), which represent such a drug delivery system, have recently attracted attention as an antigen delivery strategy [1,12,13]. LNP hold great promise in customized medicine because their manufacturing process is relatively simple and desirable properties (charge, size, surface modification, etc.) can be easily imparted by altering the composition of lipids [14,15]. It may also be possible to impart a synergistic effect by simultaneously loading an antigen and adjuvant to the LNP. In the BioNTech/Pfizer and Moderna mRNA vaccines recently approved for the prevention of coronavirus infectious disease-19 (COVID-19), LNP comprises a major component and are thought to play roles both in mRNA delivery and as an adjuvant [1].

**Figure 1:**
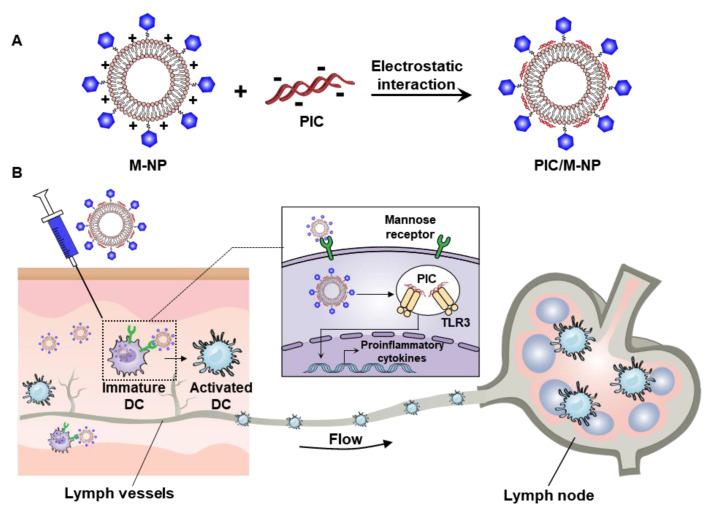
Dendritic cell **(**DC)-mediated lymph node targeting by polyinosinic:polycytidylic acid (PIC)/mannose-modified cationic lipid nanoparticle (M-NP). (**A**) PIC was complexed with M-NP via electrostatic interaction to form PIC/M-NP. (**B**) Mechanisms of DC activation and lymph node targeting by PIC/M-NP.

Here, we set out to design an LNP capable of targeting DC for lymphatic delivery of nucleic acid adjuvants. We selected polyinosinic:polycytidylic acid (poly I:C, PIC) as a model nucleic acid adjuvant. The LNP was designed to carry a cationic charge to enable the delivery of the negatively charged nucleic acid adjuvant, and mannose-modified lipid was inserted to induce DC-specific delivery. The effects of PIC-complexed mannose LNP (PIC/M-NP) on DC-specific cellular uptake and DC maturation were evaluated in vitro and in vivo (Figure 1).

## 2. Materials and Methods

### 2.1. Preparation of PIC-Loaded M-NP

M-NP were prepared using a thin-film hydration method [16]. Briefly, 0.292 μmol of 1,2-diphytanoyl-sn-glycero-3-phosphoethanolamine (DPhPE, Avanti Polar Lipids, Alabaster, AL, USA), 0.464 μmol of 3β-[*N*-(*N*’,*N*’-dimethylaminoethane)-carbamoyl]cholesterol (DC-Chol, Avanti Polar Lipids), 1.24 μmol of 1,2-dioleoyl-sn-glycero-3-ethylphosphocholine (EDOPC, Avanti Polar Lipids), and various moles of 1,2-dipalmitoyl-sn-glycero-3-phospho((ethyl-1’,2’,3’-triazole)triethyleneglycolmannose) (PA-PEG3-man-nose, PEG m.w., 148.1, Avanti Polar Lipids) were dissolved in chloroform. For flow cytometry and fluorescence microscopy, 0.03 μmol fluorescent dye cyanine 5-labeled 1,2-dioleoyl-sn-glycero-3-phosphoethanolamine-*N* (Cy5-PE, Avanti Polar Lipids) was added to the lipid mixture. The chloroform was removed, and the remaining liquid was dehydrated to a thin lipid film using a vacuum rotary evaporator. The lipid film was rehydrated with 1 mL of 20 mM 4-(2-hydroxyethyl)-1-piperazineethanesulfonic acid buffer (HEPES, pH 7.0), followed by sonication for 30 min and centrifugation at 13,500× *g* for 3 min. The supernatant was collected as M-NP. Various PA-PEG-mannose (0.04, 0.1, 0.2, or 0.4 μmol) were used to make M-NPs containing 2, 5, 10, 20 mol% mannose and described as M-NP 2, 5, 10, and 20, respectively. For the preparation of poly I:C-loaded M-NP (PIC/M-NP), 5 μg of poly I:C (average size of 0.6 kb, InvivoGen, San Diego, CA, USA) in HEPES buffer was added into 20 μL of M-NP, vortex mixing was used to form PIC/M-NP complexes via electrostatic interaction, and incubation was performed for 10 min at room temperature.

### 2.2. Characterization and Complexation Study of Nanoparticles

Nanoparticles were characterized by morphology, size, and net surface charge. The morphology of nanoparticles was measured by transmission electron microscopy (TEM) using a JEM-2100 F (JEOL, Tokyo, Japan). The size distribution and zeta potential were evaluated using an ELSZ-1000 instrument (Otsuka Electronics Co., Osaka, Japan), based on the dynamic light scattering method and laser Doppler microelectrophoresis, respectively. For size measurement, various M-NP and PIC/M-NP samples stored at 4 °C were diluted 20 mM HEPES buffer. The particle size was obtained by cumulant intensity distribution, and size was measured 3 times for each group. The particle size was measured over 1 week. The complexation of PIC and M-NP was analyzed by gel retardation assay [8]. PIC/M-NP were prepared by mixing PIC with M-NP at various weight ratios, followed by 1% agarose gel electrophoresis and analysis using a Gel Doc XR+ Imaging System (Bio-Rad, Hercules, CA, USA).

### 2.3. Animals

Five-week-old Balb/c mice were purchased from Raon Bio Korea (Yongin, Korea) and maintained under standard pathogen-free conditions. All animal experiments were performed under the Guidelines for the Care and Use of Laboratory Animals of the Institute of Laboratory Animal Resources, Seoul National University (approval number, SNU-190417-15(E)).

### 2.4. Isolation of Bone Marrow-Derived Dendritic Cells (BMDC)

BMDC were isolated as previously described [17]. Briefly, femurs and tibias were extracted from 5-week-old Balb/c mice and sterilized by immersion in 70% ethanol for 5 min. The bones were washed thrice with phosphate-buffered saline (PBS; pH 7.4), bone marrow was flushed with complete RPMI medium (Welgene, Gyeongsan, Korea), and red blood cells were lysed. The collected monocytes were resuspended in Iscove’s modified Dulbecco’s medium (Welgene) supplemented with 10% fetal bovine serum (GenDEPOT, Barker, TX, USA), 100 mg/mL streptomycin, 100 units/mL penicillin (Gibco, Carlsbad, CA, USA), 20 ng/mL recombinant mouse granulocyte-macrophage colony-stimulation factor (GenScript, Piscataway, NJ, USA), 20 ng/mL recombinant mouse interlukin-4 (GenScript) and 50 μm β-mercaptoethanol (Sigma-Aldrich, St. Louis, MO, USA). Fresh medium was added on day 3 and BMDC were ready to use on day 7. 

### 2.5. In Vitro Cytotoxicity Assay

The cell viability of nanoparticle-treated BMDC was evaluated by MTT (3-(4,5-dimethylthizol-2-yl)-2,5-diphenyltetrazolium bromide) assay (Sigma-Aldrich) and live cell staining [18]. BMDC were seeded to 24-well plates (1 × 10^5^ cells/well) and incubated for 24 h. BMDC was treated with various nanoparticle formulations at a PIC dose of 5 μg for 4 h. The medium was changed with fresh RPMI medium, and cells were incubated for 24 or 48 h and then treated with MTT (250 μg/mL) for 1 h at 37 °C. The formazan crystals in the cells were solubilized with dimethyl sulfoxide (Sigma-Aldrich), and the absorbance of the colored solution was measured using a Multi-Reader (Molecular Devices, San Jose, CA, USA) at 570 nm. The cell viability of BMDC was calculated by normalizing the absorbance of the untreated BMDC group to 100%. For live cell staining, BMDC were stained with 2 μM calcein AM (Molecular Probes, Eugene, OR, USA) for 15 min, and live BMDC was observed by a fluorescence microscope (DM IL; Leica, Buffalo Grove, IL, USA).

### 2.6. In Vitro Study of DC Targeting

The DC-targeted delivery efficiency of nanoparticles was evaluated by flow cytometry and fluorescence microscopy. For flow cytometry, BMDC were seeded in 24-well plates (1 × 10^5^ cells/well), incubated for 24 h, and treated with Cy5-labeled nanoparticles at a PIC dose of 5 μg for 4 h. The cells were harvested, washed thrice with PBS, and analyzed by flow cytometry (FACSCalibur; BD Bioscience, San Jose, CA, USA). For fluorescence microscopy, BMDC were treated with Cy5-labeled nanoparticles for 4 h, washed with PBS, and fixed with 4% formaldehyde in PBS for 10 min. The cells were then washed with PBS, stained with FITC-anti-mouse CD11c antibody (BioLegend, San Diego, CA, USA) for 1 h, and then stained with 4′,6-diamidino-2-phenylindole (DAPI, Sigma-Aldrich, St. Louis, MO, USA) for 10 min. DC-targeting images were visualized using a confocal laser-scanning microscope (LSM 5 Exciter; Carl Zeiss, Inc., Jena, Germany).

### 2.7. In Vitro Study of DC Maturation

DC maturation was evaluated by analyzing the expression level of CD86 on the cell surface [19]. BMDC were seeded in 24-well plates (1 × 10^5^ cells/well), incubated for 24 h, and treated with various nanoparticle formulations at a PIC dose of 5 μg for 24 h. The cells were harvested and stained with FITC-anti-mouse CD11c and APC-anti-mouse CD86 (BioLegend) antibodies for 1 h. For CD11c analysis, the excitation and emission wavelengths used for flow cytometry of FITC were 495 and 519 nm, respectively. For CD86 analysis, the excitation and emission wavelengths used for flow cytometry of APC were 650 and 660 nm, respectively. The expression of CD86 was analyzed using a BD FACSCalibur flow cytometer (BD Bioscience). The expression of surface markers on DC was determined by gating the CD11c+ population.

### 2.8. In Vivo Lymph Node Targeting of Nanoparticles

The in vivo lymph node targeting ability of nanoparticles was evaluated by flow cytometry and molecular imaging. Five-week-old Balb/c mice were injected subcutaneously with PIC/Cy5-labeled M-NP at a PIC dose of 0.25 mg/kg. For lymph node imaging, mice were sacrificed after 24 h and inguinal lymph nodes were extracted. The fluorescence of lymph nodes was monitored using an IVIS Spectrum in Vivo Imaging System (PerkinElmer, Waltham, MA, USA). For flow cytometry, single cells were prepared by grinding lymph nodes through 70 μM cell strainers (SPL Life Science, Pocheon, Korea) and stained with FITC-anti-mouse CD11c for 1 h. The population of DC that took up nanoparticles (CD11c^+^Cy5^+^) in the lymph nodes was measured by flow cytometry.

### 2.9. DC Maturation Study in Lymph Node

DC maturation in lymph nodes was analyzed by measuring the expression level of CD86 on DC isolated from Balb/c mice. Five-week-old Balb/c mice were injected subcutaneously in the back with various PIC-loaded nanoparticle formulations at a PIC dose of 0.25 mg/kg (5 μg PIC/mouse). After 48 h, mice were sacrificed and inguinal lymph nodes were isolated. Single-cell suspensions were stained with FITC-anti-mouse CD11c and APC-anti-mouse CD86 for 1 h. The population of activated DC (CD11c^+^CD86^+^) was analyzed by flow cytometry.

### 2.10. In Vivo Toxicity Study

Five-week-old Balb/c were injected subcutaneously in the back with various PIC-loaded nanoparticle formulations. Seven days after injection, whole blood and serum were collected for analysis of biochemical markers of organ functions. As a biomarker of liver function, alanine aminotransferase (ALT) level in the blood was measured. As a biomarker of kidney function, blood urea nitrogen (BUN) level in the blood was measured.

### 2.11. Statistics

Data were shown as mean ± standard error of the mean (SEM). The sample size (*n*) for each statistical analysis was indicated in the figure legends. A one-way analysis of variance (ANOVA) with the Student−Newman−Keuls post-hoc test was used to analyze statistical differences. The SigmaStat software (version 12.0; Systat Software, Richmond, CA, USA) was used for all statistical analysis. Significant differences are indicated as *** p < 0.001, ** p < 0.01*,* and * p < 0.05.

## 3. Results

### 3.1. Characterization of PIC/M-NP

**Figure 2:**
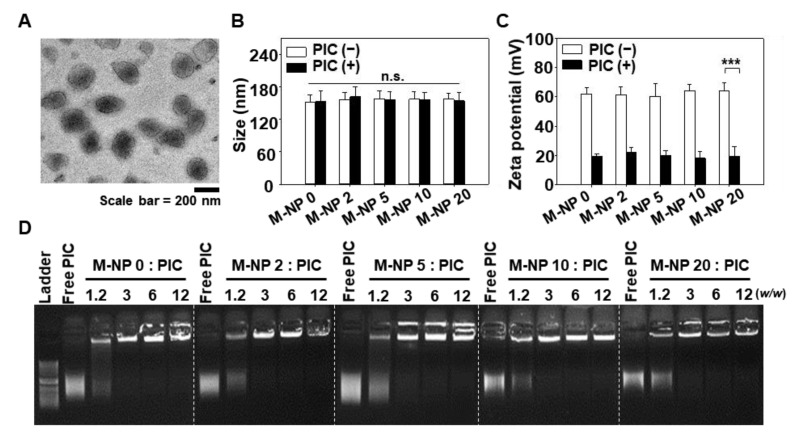
Characterization of PIC/M-NP. (**A**) Morphology of polyIC/M-NP 10 was observed by TEM. (**B**) Mean particle sizes of nanoparticles in a naked form or PIC-complexed form were measured by dynamic light scattering (*n* = 3, one-way ANOVA and Student-Newman-Keuls test). (**C**) Zeta potential was measured by laser Doppler microelectrophoresis (*n* = 3, one-way ANOVA and Student-Newman-Keuls test). (**D**) Complexation between poly IC and M-NP was determined by gel retardation. (*** *p* < 0.001, n.s.: Not significantly different).

We found that M-NP loaded sufficient PIC as a cargo regardless of the molar ratio of the targeting ligand. The physicochemical features of poly IC/M-NP were characterized with respect to morphology, size, zeta potential, and poly IC loading capacity. TEM image revealed homogenous and round morphology of PIC/M-NP 10 (Figure 2A). In size, the M-NP measured around 150 nm in diameter regardless of the mannose-PEG lipid content (Figure 2B). No significant size change was observed after lipoplex formation with PIC. The particle size of PIC/M-NP 10 was 157.7 ± 14.4 nm. The sizes of lipoplex (PIC/M-NP 10) did not significantly change over 7 days of storage at 4 °C (Figure S1). Due to the cationic lipid component, the M-NP showed a strong cationic surface charge higher than 60 mV (Figure 2C). When the cationic M-NP complexed with negatively charged poly I:C, the zeta potential decreased significantly; the zeta potential of PIC/M-NP 10 was 18.1 ± 4.2 mV. The loading capacity of poly I:C on M-NP was confirmed by gel retardation. When poly IC was complexed with M-NP at various *w*/*w* ratios, complete loading was observed at a *w*/*w* ratio of 10 (Figure 2D).

### 3.2. Cytotoxicity of PIC/M-NP

**Figure 3:**
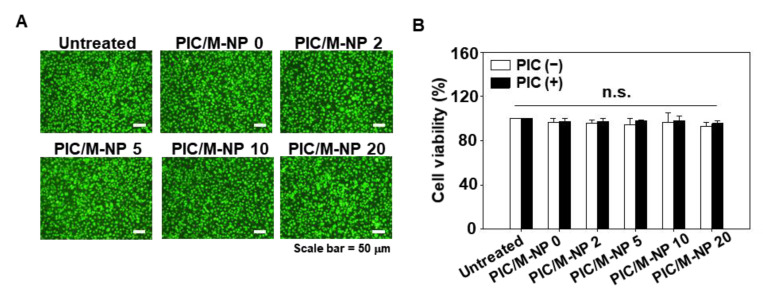
PIC/M-NP cytotoxicity on bone marrow-derived dendritic cells (BMDC). BMDC were treated with nanoparticles in a naked form or PIC-complexed form. After 24 h and 48 h, the viability of BMDC was visualized by live cell staining (**A**) and quantified by MTT assay (**B**) (*n* = 5, one-way ANOVA and Student-Newman-Keuls test). Scale bar: 50 μm. (n.s.: Not significantly different).

The poly IC/M-NP did not induce significant toxicity to BMDC. When various mannose-PEG lipid contents of PIC/M-NP were applied and cells were incubated for 24 and 48 h, no notable change was observed in live cell density (Figure 3A). Consistently, the cell viability calculated by MTT assay did not significantly differ between BMDC treated with or without PIC/M-NP for 24 or 48 h (Figure 3B).

### 3.3. Cellular Uptake of PIC/M-NP

**Figure 4:**
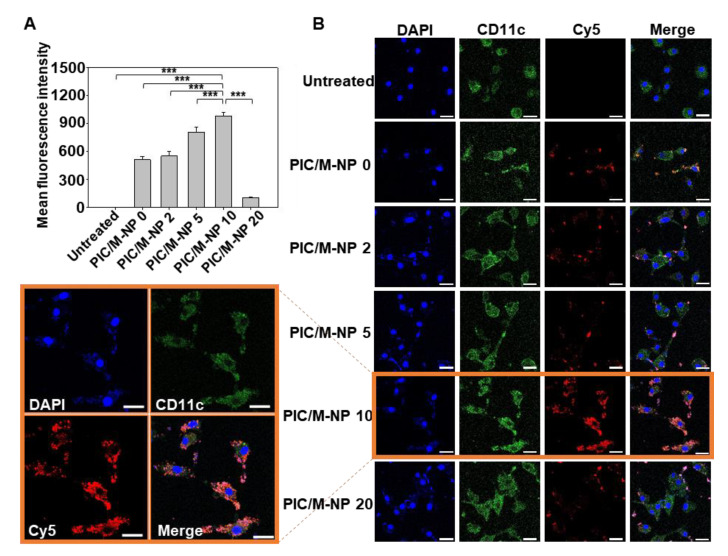
Intracellular uptake of PIC/M-NP by BMDC. BMDC were treated with PIC/M-NP with mannose densities (0 to 10%) for 4 h. (**A**) Intracellular uptake of PIC/M-NP was measured by flow cytometry (**A**). (*n* = 5, one-way ANOVA and Student-Newman-Keuls test). (**B**) Intracellular locations were visualized by confocal microscopy. Scale bar: 20 μm. (*** *p* < 0.001).

The finely controlled nanoformulation significantly improved the cellular uptake of PIC/M-NP in BMDC. As the content of mannose-PEG-lipid increased, thus did the cellular uptake of PIC/M-NP (Figure 4A). In particular, the use of 10% mannose-PEG-lipid significantly enhanced the cellular uptake, with this group showing 1.90-fold and 1.20-fold higher mean fluorescence intensity compared to those of the PIC/M-NP 0- and PIC/M-NP 5-treated groups. However, when the content of mannose-PEG-lipid was further increased to 20%, the cellular uptake was 9.35-fold lower than that obtained with PIC/M-NP 10. Consistently, confocal microscopic images revealed distinct endosomal localization of M-NP fluorescence in BMDC treated with PIC/M-NP 10, whereas this was not seen for the untreated group or those treated with PIC/M-NP of other ratios (Figure 4B).

### 3.4. In Vitro BMDC Maturation Effect of PIC/M-NP

**Figure 5:**
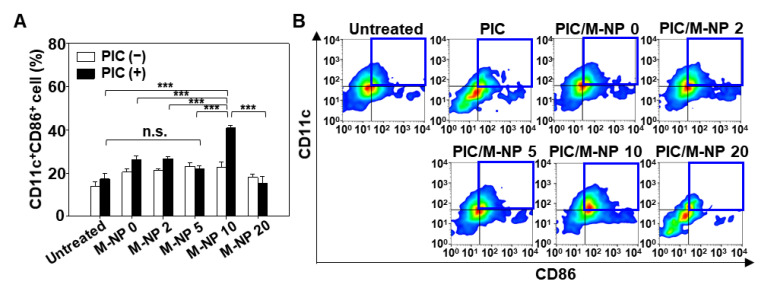
In vitro activation of BMDC by PIC/M-NP. BMDC was treated with various PIC-loaded nanoparticles. After 48 h, the expression of CD86 on BMDC was quantified by flow cytometry. (**A**) Populations of CD11c^+^CD86^+^ cells are shown for each group (*n* = 5, one-way ANOVA and Student-Newman-Keuls test). (**B**) A representative cell density plot is shown for each group. (*** *p* < 0.001). Blue boxes indicate the gated areas for CD11c+CD86+ cells.

Poly IC-loaded M-NP 10 remarkably enhanced BMDC maturation. When BMDC were treated with M-NP lacking PIC, there was no significant expression of the DC maturation marker, CD86 (Figure 5A). Similarly, free PIC treatment did not show significant increase in the maturation of BMDC. However, PIC/M-NP 10-treated BMDC showed a CD86-positive DC population of 40.7 ± 1.2%; this was significantly higher than the 13.9 ± 1.9% seen in the untreated group, indicating that PIC/M-NP 10 significantly promoted DC maturation (Figure 5B). BMDC treated with PIC/M-NP 0, which lacked the mannose-PEG-lipid, had a 26.1 ± 1.9% CD86-positive DC population, indicating that this formulation triggered DC maturation but did so less efficiently than PIC/M-NP 10.

### 3.5. In Vivo Lymph Node Targeting of PIC/M-NP

**Figure 6:**
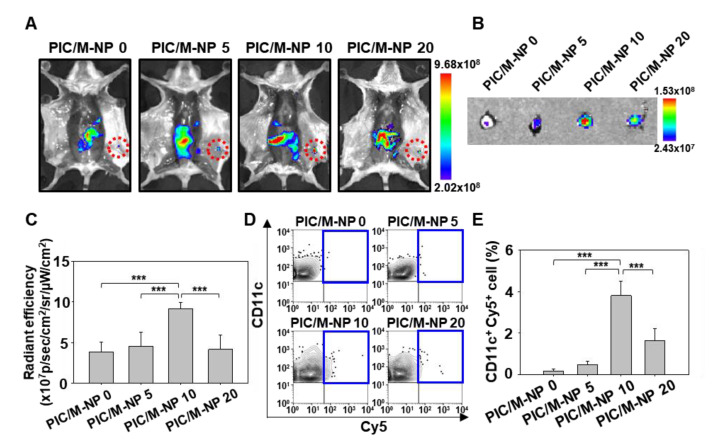
In vivo lymph node-targeting ability of PIC/M-NP. Mice were injected subcutaneously with Cy5-labeled nanoparticles. After 24 h, fluorescence at inguinal lymph node sites (**A**) and ex vivo images of inguinal lymph nodes (**B**) were visualized. (**C**) Radiant efficiency of inguinal lymph nodes was quantified for each group (*n* = 5, one-way ANOVA and Student-Newman-Keuls test). (**D**) The uptake of nanoparticles in lymph node-resident DC was measured by flow cytometry. Populations of CD11c^+^Cy5^+^ cells (**E**) are shown (*n* = 5, one-way ANOVA and Student-Newman-Keuls test, *** *p* < 0.001).

Administered PIC/M-NP exhibited greater accumulation in lymph nodes compared to the untreated group. One day after subcutaneous injection of the nanoparticle to the backs of mice, whole-body imaging revealed that mice treated with PIC/M-NP 0, poly IC/M-NP 5, or PIC/M-NP 20 showed low-intensity fluorescence signals in their inguinal lymph nodes (Figure 6A). On the other hand, higher fluorescence signal fluorescence was detected in the lymph nodes of mice treated with PIC/M-NP 10. Ex vivo images of extracted inguinal lymph nodes also showed increased PIC/M-NP 10-derived fluorescence localized in the lymph nodes (Figure 6B). The radiant efficiency calculated for the lymph nodes of the PIC/M-NP 10-treated group was 2.40-fold and 2.03-fold higher than those of the PIC/M-NP 0- and PIC/M-NP 5-treated groups, respectively (Figure 6C). Cellular uptake of PIC/M-NP was observed in the DC population of lymph nodes. The PIC/M-NP 10-treated group showed the highest DC population compared to other groups (Figure 6D), and its fluorescence positive DC population was 5.7 ± 1.0%, which was 25.7-fold higher than that seen in the PIC/M-NP 0-treated group (Figure 6E).

### 3.6. In Vivo DC Maturation Effect and Safety of PIC/M-NP

**Figure 7:**
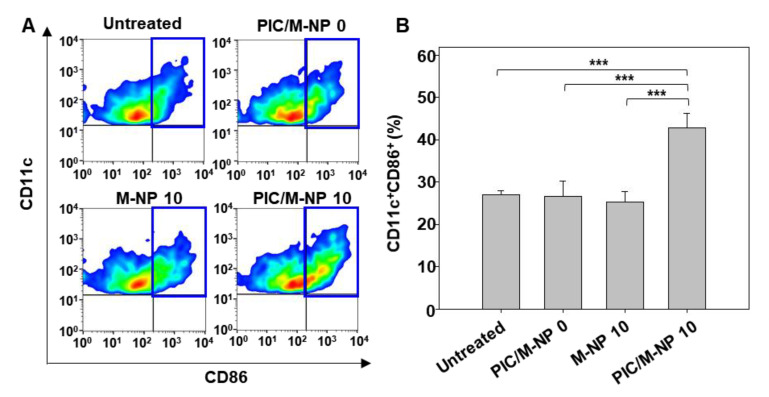
In vivo DC maturation in inguinal lymph nodes. Mice were injected subcutaneously with nanoparticles in a naked form or PIC-complexed form at a poly IC dose of 0.25 mg/kg. (**A**) After 48 h, inguinal lymph nodes were isolated, and the expression level of CD86 was measured by flow cytometry. The blue box indicates the gated area for CD11c+CD86+ cells. (**B**) Population of CD11c^+^CD86^+^ cells was quantified for each group (n = 5, one-way ANOVA and Student-Newman-Keuls test, *** *p* < 0.001).

Consistent with the enhanced cellular uptake of PIC/M-NP 10 into DC of the lymph nodes, injection of PIC/M-NP 10 into mice induced significant DC maturation, as determined by the expression of CD86. Two days after subcutaneous injection of PIC/M-NP, the CD86-positive DC population in the inguinal lymph node was analyzed. CD86-positive populations of mice were 26.7 ± 3.4% for PIC/M-NP 0-treated group, and 21.7 ± 2.6% for M-NP 10-treated group (Figure 7A). There were no significant differences in CD86-positive populations of mice between untreated (Figure 7B) and M-NP 10-treated groups. In contrast, PIC/M-NP 10 treatment efficiently induced DC maturation, yielding a positive population of 42.8 ± 3.5%, which was 1.58-fold and 1.60-fold higher than those seen in the untreated and poly IC/M-NP 0-treated groups, respectively.

**Figure 8:**
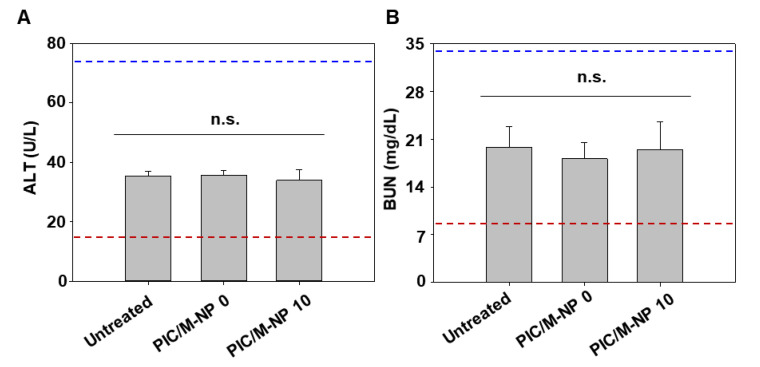
Biochemical markers of liver and kidney functions. Mice were injected subcutaneously with PIC/M-NP at a poly IC dose of 0.25 mg/kg. After 7 days, ALT (**A**) and BUN (**B**) levels in the blood were measured for liver and kidney functions, respectively. (*n* = 5, one-way ANOVA, n.s., not significant).

Subcutaneous administration of PIC/M-NP did not reveal significant toxicity. In the groups treated with PIC/M-NP 0 or PIC/M-NP 0, the levels of ALT (Figure 8A) and BUN (Figure 8B) did not significantly differ from those of the untreated group. Moreover, regardless of treatments, ALT and BUN levels were observed within normal ranges.

## 4. Discussion

In this study, we show that the developed PIC-loaded mannose conjugated nanoparticles, called PIC/M-NP, could efficiently target lymph nodes and induce immune system activation. PIC/M-NP 10, which contains an optimized 10% molar ratio of mannose-PEG-lipid in the nanoparticle, demonstrated the most efficient lymph node targeting and cellular uptake into DC, which in turn led to DC maturation by PIC.

M-NP 10 served as a suitable delivery system for introducing PIC to DC. As a synthetic analog of double-stranded RNA, poly IC is a potent immune adjuvant that can activate the innate immune system [20]. Since its receptor, Toll-like receptor 3, is mainly localized to the endosomal compartment, the PIC needs to enter the cellular endosomal pathway to be recognized [21]. However, the high molecular weight and highly negative charge of PIC hinder its penetration of the plasma membrane. Previously, PIC has been delivered using various cationic materials such as polysaccharide [22], polymer [23], and lipid [24] nanoparticles. In these studies, cationic net charges per se have not shown to induce the non-specific uptake of PIC to the lymph nodes, indicating the need for specific ligands in the design of delivery systems. In this study, the modification with mannose moieties suggested the potential of improved targeting to the lymph nodes.

In this regard, targeting mannose receptors with M-NP 10 can be an efficient strategy for delivering PIC to DC. The mannose receptor, also called CD206, is known to be overexpressed on immature DC and induce endocytosis [25]. As the major role of mannose receptors in DC is to induce internalization of pathogens by recognizing their glycans or mannosylated antigens, targeting of mannose receptors is an efficient way to deliver therapeutic agents to immune cells. [26,27]. M-NP 10 is less than 200 nm in size and thus should be suitable for receptor-mediated endocytosis (Figure 2A) [28]. The ability to load poly IC on the surface of M-NP 10 through charge-charge interaction further supports the promise of M-NP 10 as a potent delivery system for activating the immune system (Figure 2C).

It has been reported that excessive PEGylated lipids in liposomes can provide steric hindrance and reduce the efficiency of surface targeting moieties to bind to the target cells [29]. Such phenomenon was termed as ‘PEG dilemma’. To avoid the PEG dilemma, the optimization of pegylated lipid density in lipid nanoparticles would be essential. In this study, we used triethyleneglycol mannose derivative of lipid. In a previous study, di-, tetra, or hexaethylenglycol have been used to formulate mannosylated lipid nanoparticles. In the study, tetraethyleneglycol was observed to provide higher uptake by Raw 264.7 cells than diethyleneglycol or hexaethyleneglycol-based nanoparticles [30]. This study suggests the importance of PEG molecular weights in the design of nanoparticles.

The significant accumulation of signal in inguinal lymph nodes of mice subcutaneously injected with PIC/M-NP 10 indicates that the PIC/M-NP 10-internalizing DC migrated to the lymph node, where a systemic adaptive immune response can be initiated (Figure 6). Cationic nanoparticles have been reported to be taken up by macrophages in the lung, liver, and spleen [31]. A previous study reported that subcutaneous injection of nanoparticles could reduce the distribution to the liver or spleen and increase lymph node delivery compared to intravenous injection [32]. Although we observed fluorescence signals in the liver and spleen after subcutaneous injection of PIC/M-NP, biochemical markers related to liver and spleen functions remain in normal ranges (Figure 8). The uptake of PIC to DC supported their maturation and efficient migration and achieved cell-mediated lymph node targeting. As a result, significantly more activated DC were found in the lymph nodes of PIC/M-NP 10-treated mice compared to control mice and those treated with the other formulations.

The capability of PIC/M-NP 10 to efficiently target and move antigen-presenting cells to the lymph node suggest that this formulation could be a promising vaccine delivery system. As DC critically links the innate and adaptive immune systems and the lymph node is an immune-specialized organ where T and B lymphocytes are concentrated, PIC/M-NP 10 has the potential to induce profound adaptive immune responses against infectious disease or cancers.

## Data Availability

Not applicable

## Supplementary Materials

The following are available online at https://www.mdpi.com/1999-4923/13/4/490/s1, Figure S1: Stability of PIC/M-NP 10.
