# Supplementary material for: Lipid Nanoparticle-Mediated Lymphatic Delivery of Immunostimulatory Nucleic Acids
Source: Pharmaceutics. 2021 Apr 3;13(4):490. doi: 10.3390/pharmaceutics13040490 (PMC8103501; doi:10.3390/pharmaceutics13040490)
Supplement: Supplementary file 1 [file pharmaceutics-13-00490-s001.zip › pharmaceutics-1118824-SI.pdf]

pharmaceutics

MDPI

# Supplementary Materials: Lipid Nanoparticle-Mediated Lymphatic Delivery of Immunostimulatory Nucleic Acids

**Dongyoon Kim, Yina Wu, Gayong Shim and Yu-Kyoung Oh**

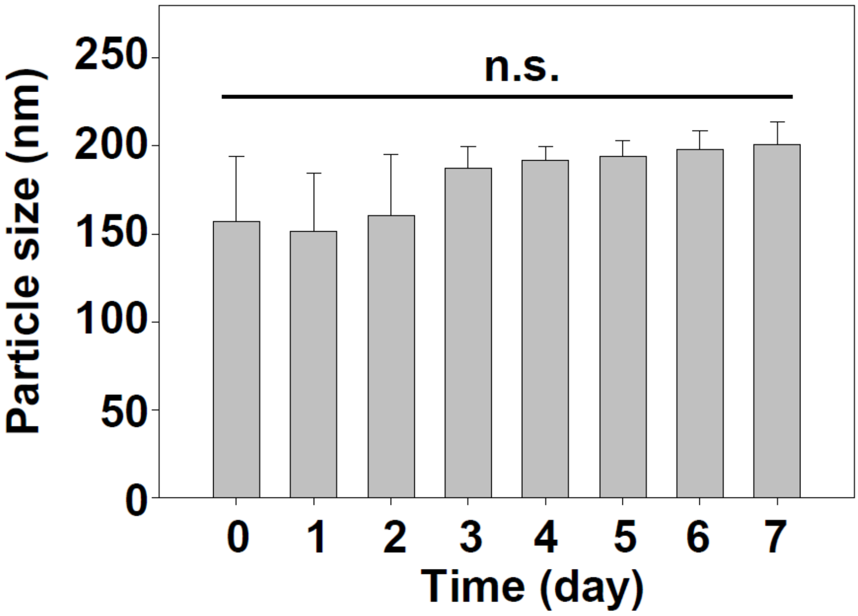


**Figure S1.** Stability of PIC/M-NP 10 (n.s.: not significantly different).

**Citation:** Kim, D.; Wu, Y.; Shim, G.; Oh, Y.-K. Lipid Nanoparticle-Mediated Lymphatic Delivery of Immunostimulatory Nucleic Acids. *Pharmaceutics* **2021**, *13*, 490. https://doi.org/10.3390/pharmaceutics13040490

**Publisher's Note:** MDPI stays neutral with regard to jurisdictional claims in published maps and institutional affiliations.

**Copyright:** © 2021 by the authors. Submitted for possible open access publication under the terms and conditions of the Creative Commons Attribution (CC BY) license (http://creativecommons.org/licenses/by/4.0/).
